# Tibetan Tea Drives *Baijiu* Flavor Formation via Microbial Niche Modulation in *Daqu*: A Multi-Omics Study

**DOI:** 10.3390/foods15142469

**Published:** 2026-07-12

**Authors:** Ting Ren, Weiqi Li, Xinyue Wen, Baichuan Hu, Zhengfeng Fang, Bin Hu, Wenjuan Wu, Zhen Zeng, Yuntao Liu

**Affiliations:** 1 College of Food Science, Sichuan Agricultural University, 46# Xinkang Road, Ya’an 625014, China; rting221@163.com (T.R.); 18200174320@163.com (W.L.); wenxinyue@stu.sicau.edu.cn (X.W.); 17360539151@163.com (B.H.); zfang@sicau.edu.cn (Z.F.); hubin2555@sina.com (B.H.); 2 College of Science, Sichuan Agricultural University, 46# Xinkang Road, Ya’an 625014, China; 72025@sicau.edu.cn

**Keywords:** Tibetan tea supplementation, *Daqu*, *Baijiu* fermentation, microbial community restructuring, flavor compounds, volatile metabolites

## Abstract

Interest in using Tibetan tea for fermented food production has increased due to its bioactive components and distinctive flavor characteristics. However, its application in *Daqu* prepared with Tibetan tea remains limited. This study investigated the effects of Tibetan tea addition on *Daqu* fermentation and *Baijiu* flavor formation using integrated microbiome and metabolome approaches. High-throughput sequencing, GC-MS, free amino acid analysis, electronic sensory analysis, and correlation network analysis were performed to characterize microbial and metabolic changes. Compared with wheat *Daqu* (WD), Tibetan tea *Daqu* (TD) showed higher microbial richness and enhanced fermentation performance (*p* < 0.05), with enrichment of functional microorganisms including *Sphingobium*, *Komagataella*, and *Cyberlindnera*, which were associated with enzyme activities and flavor precursor formation. Tibetan tea *Daqu Baijiu* (TDB) exhibited distinct metabolic profiles, with increased levels of esters, acids, terpenes, and free amino acids, contributing to a flavor profile characterized by ester aroma with sweet, umami, woody, and tea aroma characteristics. Correlation analysis revealed that Tibetan tea-driven microbial restructuring was linked to phenylalanine metabolism, esterification, and phenolic transformation pathways. These findings link raw materials, microbiota, and flavor formation, providing a basis for targeted *Baijiu* design.

## 1. Introduction

Chinese *Baijiu* is one of the world’s most renowned distilled alcoholic beverages, characterized by its unique solid-state fermentation system driven by *Daqu*, a saccharification and fermentation starter harboring complex microbial consortia. As the functional core of *Baijiu* production, *Daqu* not only supplies fermentative microorganisms but also governs the enzymatic hydrolysis of raw materials and the formation of flavor compounds through intricate microbial interactions [1]. These microorganisms secrete diverse hydrolytic enzymes, including amylases, proteases, and cellulases, which convert macromolecules in grains into fermentable substrates, while simultaneously performing key metabolic functions in the biosynthesis of aromatic compounds [2]. Consequently, *Daqu* largely determines the quality and flavor characteristics of the final fermented product [3,4].

In recent years, *Daqu* innovation has emerged as an important strategy for improving *Baijiu* quality and diversifying flavor profiles. One promising approach involves the incorporation of plant-derived materials to modulate microbial communities and metabolic activities during fermentation. Previous studies have demonstrated that the introduction of non-traditional substrates such as tartary buckwheat, peas, and green tea can significantly influence enzymatic properties, microbial succession, and flavor formation [5,6]. These plant materials are rich in bioactive compounds, including polyphenols and polysaccharides, which can exert selective pressures on microbial populations. However, current research still lacks an in-depth analysis of the mechanisms underlying plant-driven microbial community restructuring in *Daqu*, particularly systematic studies that establish correlations and causal inferences between microbial dynamics and the formation of key flavor compounds. As a post-fermented black tea, Tibetan tea differs from unfermented or semi-fermented teas in that it undergoes a microorganism-driven pile fermentation process during production, resulting in a significant restructuring of its chemical composition [7]. Unlike conventional tea materials previously applied in fermentation systems, which mainly function through their original plant-derived components, Tibetan tea contains fermentation-derived metabolites generated through microbial transformation, potentially enabling a more complex regulation of microbial succession and ecological interactions in solid-state fermentation systems. The phytochemical profile of Tibetan tea is characterized primarily by the oxidation and polymerization products of tea polyphenols (such as theabrownins) and tea polysaccharides [8,9], resulting in low astringency and a rich, mellow flavor. These compounds also possess bioactive properties including antioxidant [10], anti-obesity [11], and antihyperlipidemic effects [12]. Studies have shown that theabrownins can selectively inhibit certain microbial populations, thereby reshaping competitive relationships within the community [13]. Meanwhile, tea polysaccharides may serve as alternative carbon sources for specific microorganisms, potentially promoting niche differentiation and reshaping of the microbial community [14]. Therefore, although Tibetan tea does not act as an active fermentation inoculum, the complex chemical constituents formed during its prior fermentation may exert selective ecological pressure on microbial communities in solid-state fermentation systems. These characteristics suggest that Tibetan tea is not only a source of flavor precursors but also a potential ecological regulatory substrate capable of reshaping microbial community structure and function in *Daqu* fermentation.

Although the application of Tibetan tea in *Daqu* fermentation holds great potential, current research in this area remains limited. Therefore, this study systematically evaluates the effects of incorporating Tibetan tea into *Daqu* formulations on the quality characteristics of *Daqu*, the structure of microbial communities, and the composition of volatile and non-volatile metabolites. Furthermore, by integrating microbiome and metabolome data through co-occurrence network analysis, we elucidate the relationship between microbial community changes and the formation of flavor compounds, thereby revealing the potential mechanisms by which Tibetan tea regulates the *Daqu* fermentation system. Despite its potential, the application of Tibetan tea in *Daqu* fermentation remains largely unexplored. Critical questions remain unanswered: whether Tibetan tea can induce stable and reproducible shifts in microbial community structure, which microbial taxa are selectively enriched or suppressed, and how these changes affect the enzymatic performance of *Daqu*. More importantly, the causal linkage between Tibetan tea-induced microbial restructuring and the resulting flavor profile of *Baijiu* has yet to be elucidated. Addressing this gap is essential for advancing a mechanistic understanding of microbiota-driven flavor formation in solid-state fermentation systems.

Therefore, this study aims to investigate Tibetan tea as a functional modulator in *Daqu* innovation and to establish a mechanistic framework linking raw material intervention, microbial community restructuring, and flavor formation. Specifically, this work optimized the preparation conditions of TD, characterized microbial community shifts using high-throughput sequencing, and analyzed the flavor profile of the resulting *Baijiu* through multi-platform approaches, including electronic nose/tongue, amino acid analysis, and GC–MS (gas chromatography–mass spectrometry) (Shimadzu Corporation, Kyoto, Japan). Furthermore, microbiome and flavorome datasets were integrated through co-occurrence network analysis to elucidate microbiota-flavor associations. This work provides new insights into microbial ecology-driven fermentation control and offers a strategy for developing *Baijiu* with targeted and distinctive sensory characteristics.

## 2. Materials and Methods

### 2.1. Materials and Reagents

The primary raw materials utilized in this study were carefully selected to ensure the quality and reproducibility of the experiments. Ya’an Tibetan tea (*Camellia sinensis*, var. Meizhan), characterized by its post-fermentation attributes, was generously provided by Yingtai Tea Co., Ltd. (Ya’an, China). The tea leaves were dried at 37 °C and mechanically ground to pass through a 20-mesh sieve prior to use. Wheat (variety Xi nong 2208), serving as the main substrate for *Daqu* production, was sourced from a reputable seed company (Hefei, China). High-quality glutinous sorghum, used as the fermentation substrate for *Baijiu* production, was obtained from Shanxi Qiwesi Food Co., Ltd. (Jincheng, China). The Starter culture of *Daqu* was procured from Ruihua Biological *Daqu* Co., Ltd. (Luzhou, China), which maintains consistent microbial composition essential for traditional *Baijiu* fermentation.

All chemical reagents employed in this study were of analytical grade unless otherwise specified. Sodium hydroxide, absolute ethanol, glacial acetic acid, and concentrated sulfuric acid were purchased from Chengdu Haoboyou Technology Co., Ltd. (Chengdu, China). Biochemical reagents including Folin–Ciocalteu reagent, sulfosalicylic acid, and glucose were obtained from Chengdu Explore Biological Platform (Chengdu, China) and Shanghai Yuanye Bio-Technology Co., Ltd. (Shanghai, China). Enzymes including *α*-amylase, *α*-glucosidase, cellulase, and pectinase were acquired from Chengdu Desite Biological Technology Co., Ltd. (Chengdu, China). Standard compounds for analytical purposes, including amino acid mixed standard, 1,1-dimethyl-2-octanol, C7–C40 n-alkane mixture, and fatty acid methyl esters (FAMEs), were purchased from Shanghai Yuanye Bio-Technology Co., Ltd. (Shanghai, China) and Sigma-Aldrich (St. Louis, MO, USA).

### 2.2. Optimization of Tibetan Tea Daqu (TD) Preparation

The preparation of Tibetan Tea *Daqu* was optimized using an orthogonal design to achieve optimal fermentation performance. Wheat flour (500 g, 20 mesh) served as the base material, to which varying proportions of Tibetan tea powder were added according to the experimental design. The wheat was conditioned to an appropriate moisture content, with a soft surface and slightly firm interior, and then ground so that 30~35% of the particles passed through a 20-mesh sieve. Fermentation was carried out in a constant temperature and humidity incubator (LHS-50CH, Shanghai Yiheng Scientific Instrument Co., Ltd., Shanghai, China) at 40 °C and 85% relative humidity.

Single-factor experiments were first performed to preliminarily screen the ranges of key variables, including Tibetan tea addition, inoculum amount, moisture content, and fermentation time. Based on these results, a four-factor, three-level L9(34) orthogonal design was employed to optimize the process (Table S1). The factor levels were as follows: Tibetan tea addition (4~8%), inoculum amount (6~10%), moisture content (30~50%), and fermentation time (15~25 days). Hydrolytic activity and fermentation activity were used as evaluation indices and measured according to QB/T 4257-2011 [15].

### 2.3. Determination of the Physical and Chemical Properties of Daqu

The moisture content of matured *Daqu* was determined using the constant weight method according to the Chinese national standard method (GB/T 5009.3-2010) [16]; Total acidity was measured via acid-base titration; 10 g of *Daqu* was homogenized with 90 mL of distilled water, and the filtrate was titrated with 0.1 mol/L NaOH using phenolphthalein as indicator. Total acidity was expressed as milliliters of NaOH consumed per gram of sample. Saccharification and fermentation capacities were determined in accordance with the industry standard QB/T 4257-2011. Saccharification capacity was calculated by measuring the content of reducing sugars produced from starch hydrolysis by *Daqu*. Fermentation capacity was characterized by measuring the amount of alcohol generated during the fermentation process.

### 2.4. Fourier Transform Infrared Spectroscopy (FT-IR) Analysis

The molecular characteristics of *Daqu* samples were analyzed using FT-IR spectroscopy (Nicolet IS10, Thermo Fisher Scientific, Madison, WI, USA). Approximately 2 mg of dried *Daqu* powder was thoroughly mixed with 200 mg of spectroscopic grade KBr and pressed into a transparent pellet. Spectra were acquired in the range of 4000–400 cm^−1^ with a resolution of 4 cm^−1^ and 16 cumulative scans. Background correction was performed using a pure KBr pellet.

### 2.5. Microbial Community Analysis

Total microbial genomic DNA was extracted from *Daqu* samples using the E.Z.N.A.^®^ soil DNA Kit (Omega Bio-tek, Norcross, GA, USA) according to manufacturer’s instructions. The quality and concentration of DNA were determined by 1.0% agarose gel electrophoresis and a NanoDrop2000 spectrophotometer (Thermo Fisher Scientific, Wilmington, DE, USA) and kept at −80 °C prior to further use. The hypervariable region V3–V4 of the bacterial 16S rRNA gene were amplified with primer pairs 338F (5′-ACTCCTACGGGAGGCAGCAG-3′) and 806R (5′-GGACTACHVGGGTWTCTAAT-3′) [17] by T100 Thermal Cycler for polymerase chain reaction (PCR) amplification (BIO-RAD, Hercules, CA, USA). The PCR product was extracted from 2% agarose gel and purified using the PCR Clean-Up Kit (YuHua, Shanghai, China) according to manufacturer’s instructions and quantified using Qubit 4.0 (Thermo Fisher Scientific, Waltham, MA, USA). Purified amplicons were pooled in equimolar amounts and paired-end sequenced on an Illumina Nextseq2000 platform (Illumina, San Diego, CA, USA) according to the standard protocols by Majorbio Bio-Pharm Technology Co., Ltd. (Shanghai, China). To analyze the taxonomy of each representative 16S rRNA sequence and its corresponding gene sequence, the Ribosome Database Project (RDP) classifier algorithm was compared with the Silva database for bacteria (Release 138) and the Unite database for fungi (Release 8.0), using a confidence threshold of 70%.

### 2.6. Optimization of TDB Production

The optimized TD was employed for *Baijiu* production using traditional solid-state fermentation techniques. To improve the quality of TDB, an orthogonal experiment was conducted to optimize the production process. Five hundred grams of glutinous sorghum were soaked in distilled water at 90 °C for 12 to 24 h, steamed until no white core remained, and subsequently cooled to below 28 °C. Different proportions of TD were then added and thoroughly mixed. The mixture was piled for 24 to 48 h to promote microbial propagation and initial enzymatic reactions, followed by solid-state fermentation in a fermentation tank for 15 to 35 days.

Initially, a single-factor experiment was performed to preliminarily screen the ranges of key variables, including the amount of tea-based malt added, fermentation time, and fermentation temperature. Based on these results, a three-factor, three-level L9(3^3^) orthogonal design was employed to optimize the process (Table S2). The levels of each factor were as follows: amount of tea-based malt added (15~25%), fermentation temperature (26~30 °C), and fermentation time (20~30 days). Alcohol content was used as the evaluation criterion. After fermentation, the fermented grains were distilled using traditional Chinese steam distillation equipment. The alcohol content of the distillate was measured using an alcoholmeter according to the Chinese national standard method (GB/T 10345-2022) [18].

### 2.7. Analysis of Free Amino Acids

The fermented grains (FG) were collected after the fermentation process and dried at 30 °C to constant weight. A 0.3 g sample of the dried material was extracted with 3 mL of 5% sulfosalicylic acid solution through ultrasonication for 30 min, followed by centrifugation at 3000 rpm for 20 min. The supernatant was further centrifuged at 12,000 rpm for 30 min, and 1 mL of the resulting supernatant was filtered through a 0.22 μm membrane filter (Jiangsu Green Union Scientific Instrument Co., Ltd., Taizhou, China) for analysis.

Free amino acid composition was determined using a fully automated amino acid analyzer (L-8900, Hitachi, Tokyo, Japan) equipped with an ion-exchange column (4.6 mm × 60 mm, 3 μm) and a post-column ninhydrin derivatization system. The analysis conditions were as follows: column temperature of 40 °C, reaction temperature of 135 °C, and flow rate of 0.4 mL/min. The mobile phase consisted of lithium citrate buffers with varying pH and ionic strength. Amino acids were quantified by comparing peak areas with those of standard amino acid mixtures.

### 2.8. Analysis of Volatile Flavor Compounds

Volatile compounds in *Baijiu* samples were comprehensively analyzed using two complementary approaches. For general volatile profiling, headspace solid-phase microextraction coupled with gas chromatography–mass spectrometry (HS-SPME-GC-MS) was employed. The *Baijiu* was diluted to 5% alcohol content (*v*/*v*), and 5 mL was placed in a 20 mL SPME vial with 3 g NaCl. The sample was equilibrated at 50 °C for 10 min, followed by extraction with a 50/30 μm DVB/CAR/PDMS fiber (Supelco, Bellefonte, PA, USA) for 40 min at 50 °C with constant agitation. The extracted compounds were desorbed in the GC injector at 260 °C for 5 min in splitless mode.

GC-MS analysis was performed using an Agilent 5975C system (Agilent Technologies, Santa Clara, CA, USA) equipped with a DB-5MS capillary column (30 m × 0.25 mm × 0.25 μm). The carrier gas was helium at a constant flow rate of 1.0 mL/min. The oven temperature program was: initial temperature 60 °C held for 2 min, ramped to 200 °C at 3 °C/min, then to 260 °C at 10 °C/min and held for 5 min. The mass spectrometer was operated in electron impact (EI) mode at 70 eV, with ion source temperature of 200 °C and mass scan range of 33–450 *m*/*z*.

For comprehensive metabolomic profiling, a derivatization approach was utilized. Briefly, 100 μL of *Baijiu* sample was mixed with 400 μL of cold methanol containing ribitol as internal standard, vortexed for 30 s, and ultrasonicated in an ice bath for 10 min. After centrifugation at 13,800× *g* for 15 min at 4 °C, 350 μL of supernatant was collected and vacuum-dried. The residue was derivatized with 30 μL of methoxyamine hydrochloride (20 mg/mL in pyridine) at 80 °C for 30 min, followed by silylation with 40 μL of BSTFA (with 1% TMCS) at 70 °C for 1.5 h. The derivatized samples were analyzed by GC-MS using a DB-5MS column with the following temperature program: 50 °C for 1 min, increased to 310 °C at 8 °C/min, and held for 11.5 min.

Compounds were first tentatively matched using the NIST 14 mass spectral library and subsequently confirmed using authentic standards, resulting in final confirmation of all identified volatile compounds.

### 2.9. E-Nose and E-Tongue Analysis

The overall flavor characteristics of *Baijiu* samples were evaluated using advanced electronic sensory systems. The electronic nose (Heracles NEO, Alpha MOS, Toulouse, France) equipped with a 10-sensor metal oxide semiconductor array was used for volatile fingerprinting. Detailed descriptions of the sensors can be found in Table S3. For analysis, 15 mL of sample was placed in a 60 mL vial and equilibrated at room temperature for 15 min before measurement.

The electronic tongue (ASTREE, Alpha MOS, France) with seven cross-selective liquid sensors was employed for taste profile analysis. Detailed descriptions of the sensors can be found in Table S4. The *Baijiu* samples were diluted 5-fold with deionized water prior to analysis. Both electronic nose and tongue analyses were performed in triplicate for each sample, and the last three stable measurements were used for data analysis.

### 2.10. Statistical Analysis

All experiments were conducted with at least three independent biological replicates (*n* = 3), and data were expressed as mean ± standard deviation (SD). Statistical analysis was performed using SPSS Statistics 27.0 (IBM Corp., Armonk, NY, USA) and GraphPad Prism 9.2.0 (GraphPad Software, San Diego, CA, USA). Given the small sample size (*n* = 3) and that not all data satisfied the assumptions of normality and homoscedasticity required for parametric tests, nonparametric methods were applied. For comparisons among multiple groups, the Kruskal–Wallis test was used, followed by Dunn’s post hoc test with Bonferroni correction. A significance level of *p* < 0.05 was considered statistically significant. Orthogonal experimental data were analyzed using range analysis and variance analysis to determine optimal conditions and factor significance.

Multivariate statistical analyses, including principal component analysis (PCA) and hierarchical clustering analysis, were performed using R software (version 4.1.0) and Origin 2022 (OriginLab Corp., Northampton, MA, USA). Spearman’s rank correlation analysis was conducted to examine relationships between microbial communities and flavor compounds, with |r| > 0.6 and *p* < 0.05 considered significant. Co-occurrence network analysis was visualized using Gephi software (version 0.9.2). Sequence data processing and microbial diversity analyses were conducted using QIIME2 (version 2021.4) and the platforms provided by Biomarker Technologies Corporation.

## 3. Results

### 3.1. Optimization Results and Physicochemical Properties of TD

**Figure 1:**
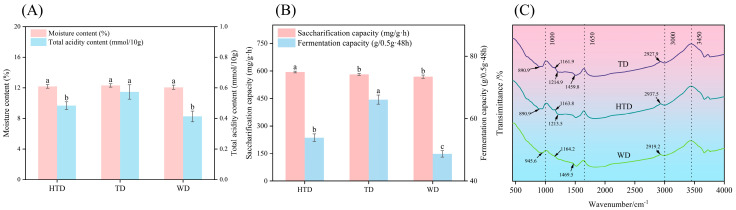
Effects of Tibetan tea addition on *Daqu*. (**A**) Moisture content and total acidity of *Daqu*; (**B**) Fermentation characteristics of *Daqu*; (**C**) FTIR spectra of *Daqu*. Different lowercase letters indicate significant differences among groups (*p* < 0.05).

As shown in Figure 1A, there was no significant difference in moisture content between TD (12.32%) and WD (12.08%), and the moisture content of all three types of *Daqu* was below the safety threshold of 13%, meeting the standards for mature *Daqu* [19]. The level of total acidity in *Daqu* is associated with organic acid metabolism driven by lactic acid bacteria and other acidogenic microbes, in addition to the decomposition of lipids, starches, and proteins [20]. The total acidity of TD (0.57 mmol/10 g) was significantly higher than that of WD (0.41 mmol/10 g). The primary reason for this may be that the addition of Tibetan tea promoted the growth and metabolism of certain acid-producing bacteria, leading to an increase in acidic metabolites and accelerated degradation of proteins, carbohydrates, and lipids, thereby generating more acidic substances. Although there was no significant difference in acidity between HTD (0.48 mmol/10 g) and WD, a slight increase was observed, suggesting that acidity may have a potential dose–response relationship with the amount added.

The extracellular enzyme activity of *Daqu* determines its fermentation performance. Saccharification capacity refers to the ability of functional microorganisms to convert starch into fermentable sugars [20]. Fermentation capacity refers to the ability of yeast to convert sugars into alcohol. As shown in Figure 1B, the saccharification and fermentation capacities of the TD reached 586.08 ± 6.41 mg/g·h and 64.01 ± 1.13 g/0.5 g·48 h, respectively. These values were significantly higher than those of WD, which were 568.47 ± 7.76 mg/g·h and 61.73 ± 0.85 g/0.5 g·48 h (*p* < 0.05), representing increases of 3.1% and 3.7%, respectively. This may be related to the introduction of Tibetan tea into the *Daqu*. *Daqu* possesses a rich enzyme system, including amylase, protease, and cellulase [21]. These complex hydrolytic enzymes serve as the driving force for the degradation of macromolecules such as starch and protein. Therefore, the addition of Tibetan tea provides a material foundation for microbial metabolism, thereby enhancing the fermentation performance of *Daqu*. The above results indicate that the addition of Tibetan tea may alter the fermentation performance of *Daqu* by influencing the microbial structure within it.

The intensity variations in FTIR absorption bands are closely associated with changes in molecular structure and chemical composition. As shown in Figure 1C, compared with the WD sample, the TD sample exhibited varying degrees of increased intensity at 1163 cm^−1^ (C-O-C stretching vibration), from 3300 to 3400 cm^−1^ (O-H stretching vibration), and at 1650 cm^−1^ (C=O stretching vibration and N H bending vibration). These spectral changes indicate significant alterations in polysaccharide structure, protein degradation, and phenolic hydroxyl content [14,22]. These spectral variations provide indirect evidence that the addition of Tibetan tea *Daqu* may have influenced the biochemical composition of the *Daqu* system.

### 3.2. Effects on the Microbial Community Composition of Daqu

**Figure 2:**
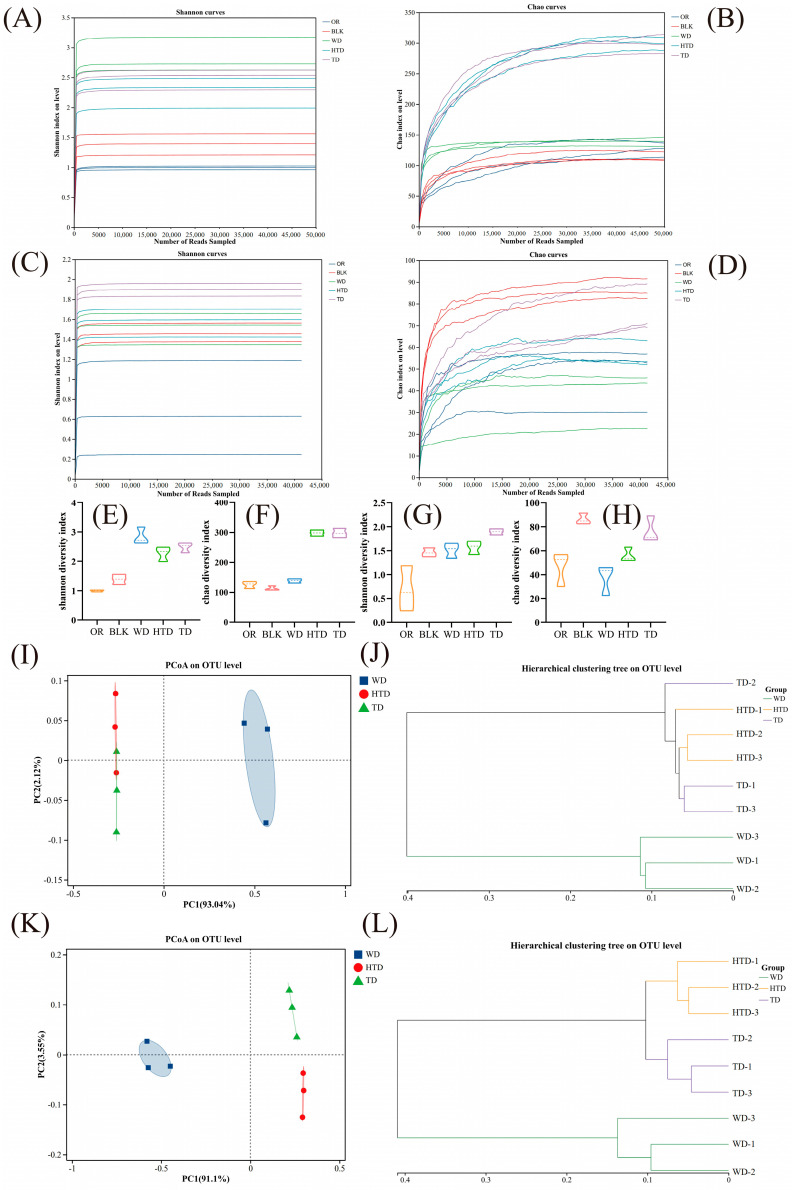
α-diversity analysis of microbial communities in *Daqu*. (**A**) Shannon index curves of bacteria; (**B**) Chao index curves of bacteria; (**C**) Shannon index curves of fungi; (**D**) Chao index curves of fungi; (**E**) Shannon index of bacteria; (**F**) Chao index of bacteria; (**G**) Shannon index of fungi; (**H**) Chao index of fungi; (**I**) PCoA plot of bacterial communities; (**J**) clustering analysis of bacterial communities; (**K**) PCoA plot of fungal communities; (**L**) clustering analysis of fungal communities. Different colors represent different sample groups. In the violin plots (**E**–**H**), the dashed lines within the violins indicate the median values of the corresponding groups.

The complex microbial community of *Daqu* plays a profound role in the flavor compound metabolism and fermentation process of *Baijiu*, thereby significantly affecting the quality and stylistic characteristics of the spirit. Therefore, elucidating the community structure and functional roles of *Daqu* microorganisms holds substantial research value [23]. Therefore, bacterial 16S rRNA and fungal ITS high-throughput sequencing were performed on the fermented samples from the TD, HTD, and WD groups. In addition, OR (original *Daqu*, serving as the mother starter culture) and BLK (blank control group) were selected as control groups to investigate and compare the regulatory effects of Tibetan tea on the *Daqu* microbial community. After processing the sequencing reads, a total of 1,105,594 raw reads were generated from 15 samples. The coverage of each sample library exceeded 98%, meeting the sequencing requirements. In Figure 2, the Shannon and Chao curves tended to plateau as the sequencing read count increased, ensuring that sufficient sequencing data were obtained to identify the microorganisms in the samples. As shown in Figure 2A–H and Tables S5 and S6, the results of the ACE index, Chao index, and Sobs index for evaluating microbial community richness, together with the Shannon and Simpson indices for assessing microbial diversity, revealed differential responses of microbial richness and diversity to Tibetan tea addition. However, both TD and HTD groups exhibited higher bacterial community richness, indicating that Tibetan tea addition mainly promoted the enrichment of bacterial species rather than increasing overall bacterial diversity. In contrast, the HTD group showed a decrease in bacterial diversity indices compared with the WD group, suggesting that Tibetan tea addition altered bacterial community diversity under the tested conditions. No significant differences in fungal community diversity were found among WD, TD, and HTD, but all three groups showed a significant increase in species richness, indicating that Tibetan tea addition primarily contributed to the enhancement of fungal community richness.

The results of the principal coordinate analysis (PCoA) and hierarchical clustering analysis presented in Figure 2I–L indicate significant differences and, more importantly, remarkably tight clustering of biological replicates within each treatment group, suggesting low within-group variation and high reproducibility of the microbial community structure among the *Daqu* samples from different treatments. For bacterial communities, the WD was clearly separated from the HTD and TD, whereas only partial separation was observed between HTD and TD. This suggests that Tibetan tea addition was the primary driving factor, while the dosage effect was relatively limited. In contrast, fungal communities exhibited a clearer separation among all groups, indicating higher sensitivity to both Tibetan tea addition and its dosage. Taken together, the tight intra-group clustering and clear inter-group divergence suggest that the observed microbial community shifts are robust and stable, with fungal communities being more responsive to environmental changes.

**Figure 3:**
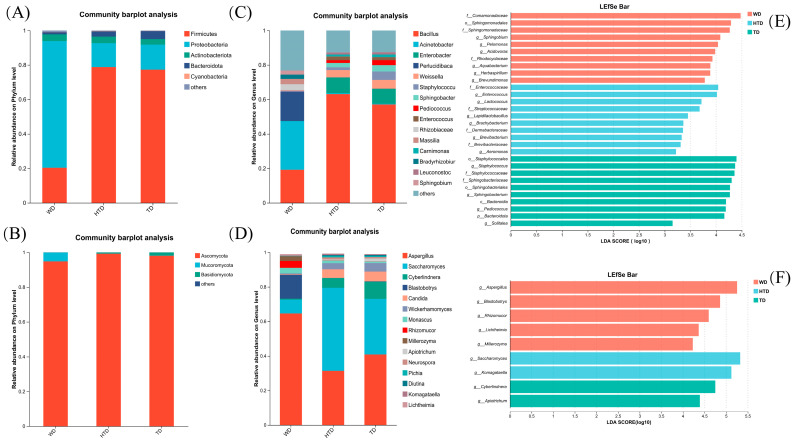
Composition of microbial communities in *Daqu*. (**A**) Bacterial composition at the phylum level; (**B**) Fungal composition at the phylum level; (**C**) Bacterial composition at the genus level; (**D**) Fungal composition at the genus level; (**E**) LEfSe analysis of bacterial communities; (**F**) LEfSe analysis of fungal communities.

Figure 3 reveals changes in the composition of the microbial community in *Daqu*. As shown in Figure 3A,B, at the phylum level, Proteobacteria and Mucoromycota were more abundant in WD, while Firmicutes, Bacteroidota, and Basidiomycota were significantly enriched in the TD and HTD groups. At the genus level (Figure 3C,D), compared to WD, the genera *Saccharomyces*, *Cyberlindnera*, *Candida*, *Wickerhamomyces*, *Apiotrichum*, *Neurospora*, *Pichia*, and *Diutina* were higher in the HTD and TD groups compared to the WD group. Previous studies have demonstrated that *Saccharomyces* and *Pichia* are associated with ester production, particularly ethyl hexanoate formation, and are considered important aroma-related microorganisms during *Baijiu* fermentation [24]. Therefore, the enrichment of these yeasts in HTD and TD may be associated with ester-related flavor development.

The LEfSe algorithm was then performed to identify the enriched features in specific types of TD. The results showed that, within the bacterial community (Figure 3E), genera exhibiting significant differences in HTD and TD included *Comamonadaceae*, *Blastobotrys*, *Pelomonas*, *Aquabacterium*, and *Sphingobacterium*, among others. Studies have shown that *Sphingobacterium* is associated with cellulose degradation, and it may release fiber components from tea, thereby enhancing the complex aromas of the liquor [25,26]. In the present study, the enrichment of *Sphingobacterium* in TD and HTD suggests its potential association with plant substrate utilization during fermentation. In the fungal community (Figure 3F), the genera showing significant differences between HTD and TD included *Komagataella*, *Apiotrichum*, and *Cyberlindnera*, which were respectively associated with the utilization of non-conventional carbon sources [27], the degradation of complex plant-derived substrates and precursor release [28], and aroma-related biotransformation [29]. Their enrichment in TD and HTD suggests their potential involvement in substrate utilization and flavor-related processes.

### 3.3. Effects on the Free Amino Acid Content in Fermented Grains

**Figure 4:**
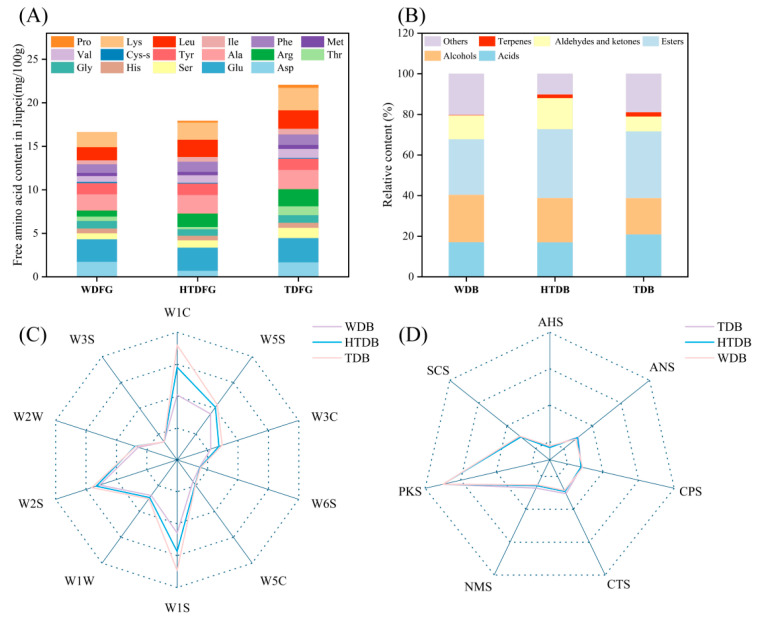
Effects of Tibetan tea addition on flavor compounds in *Baijiu*. (**A**) Content of free amino acids in *Baijiu*; (**B**) Relative content of volatile compounds in *Baijiu*; (**C**) Electronic nose analysis; (**D**) Electronic tongue analysis.

*Baijiu* fermentation is a complex microbial metabolic process in which raw materials are degraded by various microorganisms, producing large amounts of fermentable substances such as proteins, amino acids, and short peptides. Among these, free amino acids serve as precursors for volatile compounds such as higher alcohols, aldehydes, ketones, and esters, thereby directly or indirectly influencing the aromatic components of *Baijiu*. As shown in Figure 4A, the fermented grains contain a wide variety of amino acids at relatively high concentrations, and significant differences in amino acid profiles were observed among the sample groups. The total free amino acid content in the TDFG was significantly higher than that in the other two groups, with glutamic acid (Glu), alanine (Ala), and proline (Pro) being the most abundant. Glu is a typical umami amino acid that may participate in the Maillard reaction during subsequent fermentation and aging to generate complex flavor compounds such as nutty aromas [30,31]. Ala and Pro contribute to a slightly sweet taste and mouthfeel and may also act as potential precursors for aroma formation. The increased free amino acid content observed in TDFG may be associated with changes in the fermentation environment induced by the addition of Tibetan tea *Daqu*. However, the specific mechanisms underlying this phenomenon remain unclear. These changes may involve microbial activity shifts and alterations in substrate utilization during fermentation, but further studies are required to clarify the underlying biochemical and microbial processes.

### 3.4. Effects on Volatile Compounds in Baijiu

Using gas chromatography and mass spectrometry (GC MS) coupled with solid-phase microextraction (SPME) and BSTFA derivatization, a total of 239 characteristic flavor compounds were identified across three sets of *Baijiu* samples. Their relative abundances by category are shown in Figure 4B. Most esters are aromatic volatile compounds and constitute the primary components of *Baijiu* aroma [32]. In TDB, the levels of esters such as phenethyl acetate, cinnamyl acetate, diethyl succinate, and ethyl 9 decenoate were elevated. Combined with the microbial data discussed earlier, it is speculated that the genera *Komagataella* and *Cyberlindnera* may have enriched the ester profile through sugar metabolism, imparting floral and fruity aromas to the liquor. Although aldehydes and ketones decreased overall, distinct flavor compounds such as nonanal and citral increased. This is likely due to the Comamonadaceae family generating medium-chain aldehydes (e.g., nonanal) via the beta-oxidation pathway, which imparts a grassy aroma. As characteristic components of Tibetan tea, protocatechuic acid and linalool led to an increase in phenolic and terpenoid compounds in the Tibetan tea-infused liquor, endowing the *Baijiu* with the unique woody and aged aromas characteristic of Tibetan tea.

**Figure 5:**
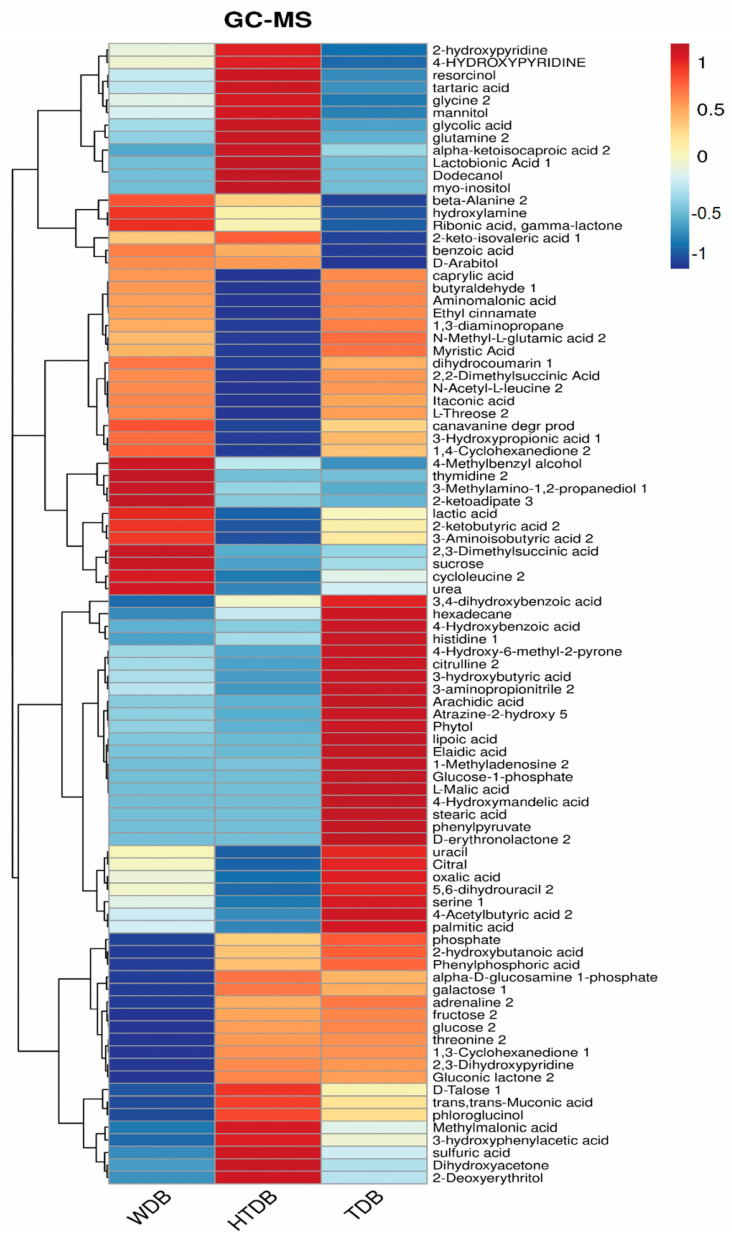
Major differential flavor compounds in *Baijiu*.

Subsequently, a cluster heatmap analysis was performed on the three sample groups, as shown in Figure 5. The HTDB group significantly enhanced the fruity and sweet aroma characteristics of the *Baijiu* through the addition of esters such as ethyl butyrate. Although the addition of ethyl benzoate increased the complexity of the aroma, the content of these esters was low, resulting in a limited impact on the overall flavor. The relative content of alcohols decreased in both the HTDB and TDB groups, with a significant reduction in higher alcohols and related compounds. Acids not only influence the flavor and mouthfeel of *Baijiu* but also enhance its sweetness, eliminate bitterness and off flavors, and increase its richness and aftertaste. In HTDB, the primary acidic compounds that differed were aromatic acids such as phenylpyruvic acid and alpha ketoisocaproic acid, which can be converted into floral and fruity compounds like phenethyl alcohol and isopentanol. Additionally, other differential compounds, including nonanoic acid and arachidic acid, contribute a waxy aroma to the spirit. The results from the e-nose and e-tongue (Figure 4C,D) are consistent with the free amino acid findings, indicating that the addition of Tibetan tea enhances the freshness, sweetness, and aromatic compound profile of TDB.

### 3.5. Co-Occurrence Network Between Microbiota and Flavor Compounds

**Figure 6:**
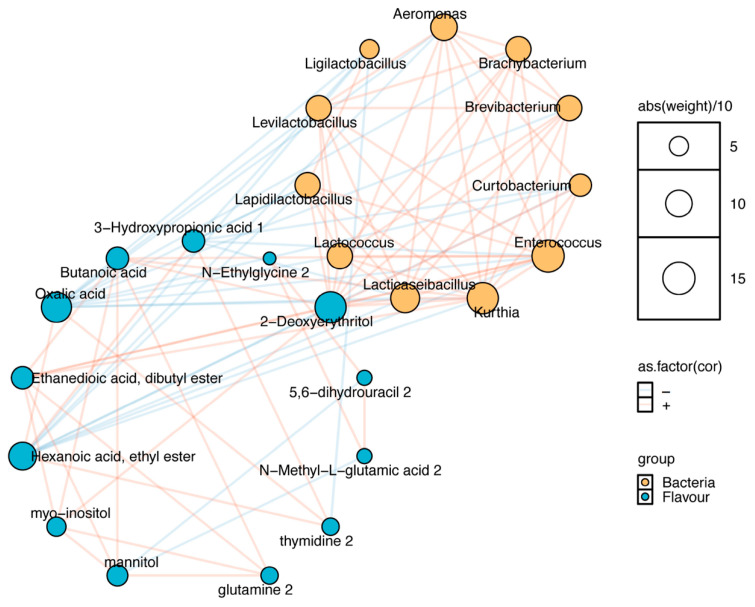
Spearman’s correlation heatmap between HTD-enriched microbial genera (LDA > 3.0, *p* < 0.05) and HTDB-characteristic flavor compounds (VIP > 1.0, *p* < 0.05). Red and blue squares indicate positive and negative correlations, respectively. Only correlations with |r| > 0.6 and *p* < 0.05 are shown.

**Figure 7:**
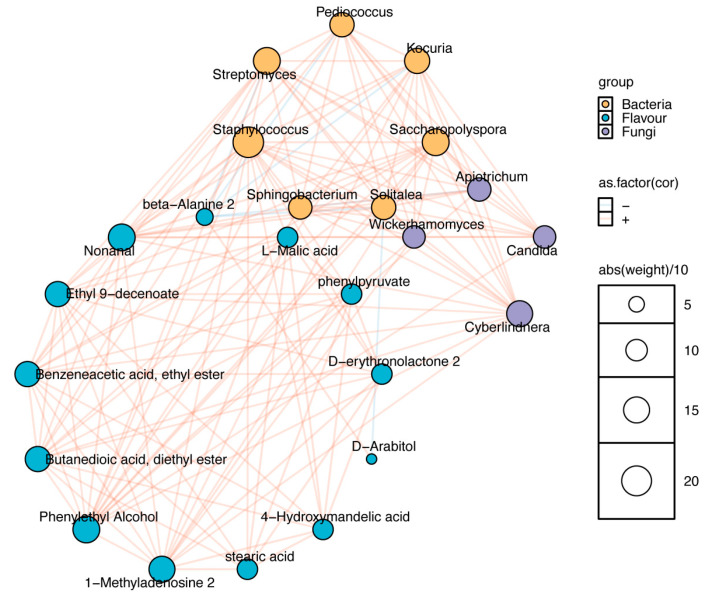
Spearman’s correlation heatmap between TD-enriched microbial genera (LDA > 3.0, *p* < 0.05) and HTDB-characteristic flavor compounds (VIP > 1.0, *p* < 0.05). Red and blue squares indicate positive and negative correlations, respectively. Only correlations with |r| > 0.6 and *p* < 0.05 are shown.

Co-occurrence network analysis revealed intricate relationships between TD-enriched microorganisms and characteristic flavor compounds in TB (Figure 6 and Figure 7). The analysis demonstrated *Sphingobium* exhibited strong positive correlations with phenylpyruvate (r = 0.86, *p* < 0.001) and phenethyl alcohol (r = 0.82, *p* < 0.001), suggesting a potential association with phenylalanine-related metabolic pathways. Similarly, *Komagataella* showed significant associations with various ester compounds including phenethyl acetate (r = 0.79, *p* < 0.001) and ethyl cinnamate (r = 0.75, *p* < 0.001), indicating possible associations with ester-related flavor formation. The network analysis identified several microbial clusters potentially associated with the flavor profile of TB. One prominent cluster centered around *Staphylococcus* and *Candida* showed strong interconnections (r = 0.84, *p* < 0.001) and joint correlations with phenethyl acetate (r = 0.81 and r = 0.78 respectively, *p* < 0.001). Another significant cluster involved *Streptomyces*, *Pediococcus*, and *Kocuria*, which collectively correlated with multiple flavor compounds including nonanal (green, citrus, average r = 0.73 ± 0.04) and L-malic acid (refreshing acidity, average r = 0.69 ± 0.05), suggesting potential associations with flavor-related chemical profiles. Notably, several TD-enriched microorganisms showed negative correlations with compounds characteristic of traditional *Baijiu*. Ethyl hexanoate demonstrated negative associations with *Ligilactobacillus* (r = −0.72, *p* < 0.01), *Aeromonas* (r = −0.68, *p* < 0.01), and *Kurthia* (r = −0.65, *p* < 0.01). Overall, the network analysis identified 34 significant microbe–flavor correlations (|r| > 0.6, *p* < 0.05), suggesting correlation-based associations that may contribute to the distinct flavor characteristics of TB.

## 4. Discussion

This study provides compelling evidence that Tibetan tea addition reshapes the *Daqu* microbial ecosystem and directs the formation of distinctive flavor compounds in *Baijiu*. The enrichment of bacterial genera such as *Sphingobium*, *Comamonadaceae*, and *Pelomonas*, together with yeasts including *Komagataella* and *Cyberlindnera*, suggests that Tibetan tea exerts selective pressure on microbial community assembly. Similar shifts in microbial composition induced by plant-derived substrates rich in polyphenols and polysaccharides have been reported in other fermentation systems [33,34,35]. However, unlike previous studies focusing on direct flavor contributions, the present work highlights a substrate-driven ecological regulation mechanism, in which Tibetan tea indirectly governs flavor formation by reshaping microbial community structure. These microbial changes were positively associated with enhanced saccharification and fermentation power, which is consistent with previous findings that community restructuring can improve enzymatic efficiency in solid-state fermentation [5].

The flavor profile of TB differs markedly from that of WB and appears to be driven by three major metabolic transformations. By integrating differential metabolite profiling with correlation network analysis between microbes and metabolites, which respectively capture the key altered metabolites and their potential microbial drivers, three major metabolic pathways were identified as central mechanisms underlying flavor differentiation. First, the enhancement of phenylalanine metabolism is supported by the strong association between *Sphingobium* and intermediates such as phenylpyruvate and phenethyl alcohol. Phenylalanine-derived pathways are widely recognized as key contributors to floral aroma formation in fermented beverages [36,37]. Although *Sphingobium* is primarily known for aromatic compound degradation, recent studies suggest its involvement in complex aromatic metabolism, potentially contributing to the formation of higher alcohols and related volatiles [38]. Therefore, the observed correlation may indicate a previously underexplored role of this genus in *Baijiu* aroma formation, although direct functional validation is still required.

Second, esterification metabolism was notably enhanced, with *Komagataella* and *Cyberlindnera* showing strong associations with multiple ester compounds. Yeasts from these genera have been reported to possess efficient ester-forming capabilities and active alcohol acetyltransferase systems [39,40,41]. Their enrichment may therefore explain the increased accumulation of fruity and floral esters in TB, suggesting that yeast-mediated esterification plays a dominant role in flavor diversification. Similar observations have been reported in *Baijiu* and other fermented systems, where yeast community composition strongly influences ester profiles [42].

Third, phenolic transformation pathways appeared to be significantly modified, as indicated by increased levels of protocatechuic acid and related compounds. *Sphingobium* species are known for their ability to degrade lignin-derived compounds [43], suggesting a plausible role in converting Tibetan tea polyphenols into volatile phenolic compounds. This transformation may contribute to the woody and complex aroma notes observed in TB. However, rather than representing a direct transfer of tea-derived compounds, these results likely reflect microbial-mediated biotransformation processes, as previously described in fermented tea and cereal systems. The observed microbial community restructuring further suggests that Tibetan tea components may function as ecological modulators. Polyphenols are known to exert selective antimicrobial effects, while polysaccharides can act as alternative carbon sources, thereby reshaping microbial niches [44,45]. In this context, Tibetan tea may create ecological conditions favoring tannin-tolerant and fiber-degrading microorganisms, while inhibiting certain traditional *Baijiu*-associated taxa. This selective pressure provides a plausible explanation for the reduction in conventional flavor compounds and the emergence of a distinct flavor profile.

Co-occurrence network analysis further revealed potential synergistic interactions among microbial taxa involved in flavor biosynthesis. For example, the association between the Staphylococcus-Candida cluster and phenethyl acetate production suggests cooperative metabolic interactions. Such cross-feeding and co-metabolism mechanisms have been widely reported in complex fermentation systems and are considered critical for flavor diversification [46,47,48]. These findings extend current understanding beyond simple correlation analyses and suggest the presence of cooperative metabolic modules underlying flavor biosynthesis, highlighting the importance of microbial consortia in shaping *Baijiu* flavor complexity.

From an applied perspective, this study demonstrates the feasibility of modulating fermentation outcomes through targeted microbial community engineering using functional raw materials. Unlike traditional approaches relying on starter optimization or enzyme supplementation, this strategy leverages substrate-driven ecological selection. Unlike previous studies using raw plant materials, this study leverages a pre-fermented tea (Tibetan tea), whose complex chemical profile is already shaped by microbial transformation during pile-fermentation. This represents a novel prebiotic-like strategy distinct from direct addition of unfermented botanicals. This strategy also offers a potentially scalable approach for industrial *Baijiu* production to achieve targeted flavor profiles without major modifications to traditional fermentation processes.

Nevertheless, several limitations should be acknowledged. The specific Tibetan tea-derived compounds responsible for microbial selection remain unclear and require targeted metabolomic identification. In addition, the causal relationships between microbial taxa and flavor compound formation need to be validated through controlled fermentation experiments and functional genomics approaches. Furthermore, the stability and reproducibility of the restructured microbial community under industrial conditions warrant further investigation.

In conclusion, this study demonstrates that Tibetan tea influences *Baijiu* fermentation through microbial community restructuring and metabolic pathway modulation. These findings establish a novel framework in which functional raw materials act as ecological regulators, providing new insights into substrate-driven microbial engineering in solid-state fermentation.

## 5. Conclusions

This study optimized both the preparation process of Tibetan tea *Daqu* and the fermentation conditions of TDB to enhance *Daqu* fermentation performance and improve *Baijiu* quality. This study introduces a paradigm shift from ingredient-based flavoring to microbiota-directed ecological engineering. Tibetan tea addition increased saccharification and fermentation capacities, altered the biochemical composition of *Daqu*, and reshaped the microbial community by enriching functional bacteria and yeasts. These changes promoted amino acid accumulation and redirected flavor metabolism. Integrated metabolomic and correlation network analyses indicated that phenylalanine metabolism, esterification, and phenolic transformation were key pathways underlying the formation of the characteristic floral, fruity, and woody aromas of TDB. These findings demonstrate that Tibetan tea can serve as both a functional substrate and an ecological regulator, offering a promising strategy for process optimization and quality improvement in *Baijiu* fermentation. From an application perspective, this study demonstrates that targeted flavor modulation can be achieved without genetic modification or exogenous enzyme addition, simply by introducing a functionally designed *Daqu* ingredient (Tibetan tea). This offers a low-regulatory-barrier strategy for *Baijiu* producers seeking flavor differentiation.

## Data Availability

The original contributions presented in this study are included in the article/Supplementary Material. Further inquiries can be directed to the corresponding authors.

## Supplementary Materials

The following supporting information can be downloaded at: https://www.mdpi.com/article/10.3390/foods15142469/s1, Table S1: Orthogonal experimental design, optimization, and validation of TD preparation conditions; Table S2:Orthogonal experimental design and optimization of fermented TDB preparation conditions; Table S3: Electronic nose sensors and its corresponding representative sensitive compounds; Table S4: Electronic tongue sensors and their corresponding representative sensitive compounds; Table S5: Alpha diversity of bacterial communities in samples from different treatment groups; Table S6: Alpha diversity of fungal communities in samples from different treatment groups.

## Abbreviations

TD	Tibetan tea *Daqu*
HTD	Half-dose Tibetan tea *Daqu*
WD	Wheat *Daqu*
TDFG	The fermented grains prepared using Tibetan tea *Daqu*
HTDFG	The fermented grains prepared using half-dose Tibetan tea *Daqu*
WDFG	The fermented grains prepared using Wheat *Daqu*
TDB	Tibetan tea *Daqu Baijiu*
HTDB	Half-dose Tibetan tea *Daqu Baijiu*
WDB	Wheat *Daqu Baijiu*
Asp	asparagine
Ala	alanine
Arg	arginine
Cys	cysteine
Glu	glutamic acid
Gly	glycine
His	histidine
Pro	proline
Ser	serine
Tyr	tyrosine
Ile	isoleucine
Leu	leucine
Lys	lysine
Met	methionine
Thr	threonine
Phe	phenylalanine
Val	valine
Thea	theanine
Trp	tryptophan
FTIR	Fourier Transform Infrared Spectroscopy
GC-MS	gas chromatography and mass spectrometry
E-nose	electronic nose
E-tongue	electronic tongue
PCoA	principal coordinates analysis
PCA	Principal Component Analysis
HS-SPME-GC-MS	headspace solid-phase microextraction coupled with gas chromatography–mass spectrometry
BSTFA	N,O-Bis(trimethylsilyl)trifluoroacetamide
TMCS	Trimethylchlorosilane
LDA	Linear Discriminant Analysis
VIP	Variable Importance in Projection
