# Supplementary material for: Tibetan Tea Drives Baijiu Flavor Formation via Microbial Niche Modulation in Daqu: A Multi-Omics Study
Source: Foods. 2026 Jul 12;15(14):2469. doi: 10.3390/foods15142469 (PMC13409105; doi:10.3390/foods15142469)
Supplement: Supplementary file 1 [file foods-15-02469-s001.zip › foods-4413988-supplementary.pdf]

Supplementary Materials

Table S1 Orthogonal experimental design, optimization, and validation of TD preparation conditions

Table S1-1 Orthogonal Experimental Factor Levels for the Preparation of TD

	A<br>Tibetan Tea Addition<br>(%)	B<br>Starter culture of *Daqu*<br>(%)	C<br>Water Addition<br>(%)	D<br>Fermentation Time<br>(day)
Level 1	4	6	30	15
Level 2	6	8	40	20
Level 3	8	10	50	25

Table S1-2 Orthogonal experimental results of TD preparation

Experimental No.	A	B	C	D	Saccharifying Power (mg/g·h)	Fermentation Power (g/0.5 g·48 h)
1	1	1	1	1	568.47	61.73
2	1	2	2	2	547.52	65.41
3	1	3	3	3	521.3	58.36
4	2	1	2	3	615.57	62.19
5	2	2	3	1	582.47	65.87
6	2	3	1	2	573.8	60.93
7	3	1	3	2	547.52	65.87
8	3	2	1	3	603.67	62.86
9	3	3	2	1	530.62	57.21
K1	1637.29	1731.56	1745.94	1681.56		
K2	1771.84	1733.66	1693.71	1668.84		
K3	1681.81	1625.72	1651.29	1740.54		
k1	545.76	577.19	581.98	560.52		
k2	590.61	577.89	564.57	556.28		
k3	560.60	541.91	550.43	580.18		
R	44.85	35.98	31.55	19.66		
K1	185.50	189.79	185.52	184.81		
K2	188.99	194.14	184.81	192.21		
K3	185.94	176.50	190.10	183.41		
k1	61.83	63.26	61.84	61.60		
k2	63.00	64.71	61.60	64.07		
k3	61.98	58.83	63.37	61.14		
R	1.02	4.43	1.76	2.93		

Table S1-3 Verification Experiment Results

Experimental combination	Saccharifying Power (mg/g·h)	Fermentation Power (g/0.5 g·48 h)
	586.71	64.41
$A_2B_2C_2D_2$	579.38	62.73
	592.16	64.88
	583.24	60.16
$A_2B_2C_2D_3$	587.15	57.29
	566.39	57.65

Table S2 Orthogonal experimental design and optimization of fermented TDB preparation conditions

Table S2-1 Orthogonal experimental factor levels for the preparation of fermented TDB

	A	B	C
	TD Addition(%)	Fermentation Temperature(℃)	Fermentation Time(day)
Level 1	15	26	20
Level 2	20	28	25
Level 3	25	30	30

Table S2-1 Orthogonal experimental results for the preparation of TDB

	A	B	C	D	Alcohol Content/（°）
1	1	1	1	1	31.4
2	1	2	2	2	34.8
3	1	3	3	3	30.1
4	2	1	2	3	29.7
5	2	2	3	1	33.4
6	2	3	1	2	34.8
7	3	1	3	2	28.6
8	3	2	1	3	30.2
9	3	3	2	1	33.5
K1	96.30	89.70	96.40	98.30	
K2	97.90	98.40	98.00	98.20	
K3	92.30	98.40	92.10	90.00	
k1	32.10	29.90	32.13	32.77	
k2	32.63	32.80	32.67	32.73	
k3	30.77	32.80	30.70	30.00	
R	1.87	2.90	1.97	2.77	

Table S3

Electronic nose sensors and its corresponding representative sensitive compounds

Sensors	Sensitive compounds
W1C	Aromatic compounds (e.g., benzene, toluene)
W5S	Nitrogen oxides (NOx)
W3C	Ammonia and aromatic compounds
W6S	Hydrogen (H_2)
W5C	Alkanes and aromatic compounds (short-chain hydrocarbons)
W1S	Methane (CH_4) and broad-range hydrocarbons
W1W	Sulfur-containing compounds (e.g., H_2S)
W2S	Alcohols (especially ethanol) and partially aromatic compounds
W2W	Aromatic compounds and organic sulfur compounds
W3S	Long-chain alkanes

Table S4

Electronic tongue sensors and their corresponding representative sensitive compounds

Sensors	Taste attribute	Representative sensitive compounds
AHS	Sourness	Organic acids (e.g., lactic acid, acetic acid, citric acid)
CTS	Saltiness	Inorganic salts (e.g., NaCl, KCl, ionic compounds)
NMS	Umami	Amino acids and nucleotides (e.g., monosodium glutamate, IMP, GMP)
PKS	Broad sensitivity	Bitter compounds and complex organics (e.g., alkaloids, polyphenols)
CPS	Broad sensitivity	Nitrogen-containing compounds (e.g., caffeine, pyrazines)
ANS	Broad sensitivity	Aldehydes and esters (e.g., hexanal, ethyl acetate)
SCS	Broad sensitivity	Earthy/musty compounds (e.g., geosmin, 2-methylisoborneol)

Table S5

Alpha diversity of bacterial communities in samples from different treatment groups

Sample\Estimators	Ace	Chao	Coverage	Shannon	Simpson	Sobs
OR	146.40±30.44b	125.90±11.83bc	0.9995±0.0001d	0.99±0.03c	0.46±0.01a	102.67±17.21c
BLK	116.37±9.66b	113.51±7.69c	0.9997±0.0000b	1.39±0.18c	0.43±0.09a	107.33±7.57c
TD	307.24±19.16a	298.23±15.61a	0.9990±0.0002c	2.49±0.17ab	0.23±0.04bc	263.00±13.53a
HTD	314.44±18.27a	298.53±10.36a	0.9989±0.0001d	2.27±0.25b	0.28±0.06b	261.00±10.82a
WD	137.99±5.69b	138.84±7.41b	0.9999±0.0001a	2.84±0.29a	0.13±0.03c	136.33±4.62b

a-d: Mean value in the same column with different letters was significantly different ($P < 0.05$) by ANOVA and Duncan's test. OR, original feces; BLK, Blank control; TD, Tibetan tea *Daqu*; HTD, Half dose Tibetan tea *Daqu*; WD, Wheat *Daqu*.

Table S6

Alpha diversity of fungal communities in samples from different treatment groups

Sample\Estimators	Ace	Chao	Coverage	Shannon	Simpson	Sobs
OR	48.84±16.13c	46.55±14.48c	0.9999±0.0001ab	0.69±0.47b	0.71±0.22a	43.67±13.05c
BLK	86.68±4.07a	86.29±4.67a	0.9998±0.0000ab	1.46±0.09a	0.48±0.03b	83.67±4.51a
TD	78.95±20.25ab	76.45±11.00ab	0.9997±0.0001b	1.9±0.06a	0.22±0.01c	64.00±8.72b
HTD	58.82±6.16bc	56.08±6.03bc	0.9998±0.0001ab	1.57±0.14a	0.32±0.04bc	54.33±5.13bc
WD	38.44±12.93c	37.29±12.86c	0.9999±0.0000a	1.52±0.16a	0.38±0.04bc	36.33±13.32c

a-d: Mean value in the same column with different letters was significantly different ($P < 0.05$) by ANOVA and Duncan's test. OR, original feces; BLK, Blank control; TD, Tibetan tea *Daqu*; HTD, Half dose Tibetan tea *Daqu*; WD, Wheat *Daqu.*
